# Meta-analysis of a 10-plex urine-based biomarker assay for the detection of bladder cancer

**DOI:** 10.18632/oncotarget.23872

**Published:** 2018-01-03

**Authors:** Norihiko Masuda, Osamu Ogawa, Meyeon Park, Alvin Y. Liu, Steve Goodison, Yunfeng Dai, Landon Kozai, Hideki Furuya, Yair Lotan, Charles J. Rosser, Takashi Kobayashi

**Affiliations:** ^1^ Department of Urology, Kyoto University Graduate School of Medicine, Kyoto 606-8507, Japan; ^2^ Department of Medicine, University of California San Francisco, San Francisco, CA 94143, USA; ^3^ Department of Urology, University of Washington, Seattle, WA 98195, USA; ^4^ Department of Health Sciences Research, Mayo Clinic, Jacksonville, FL 32224, USA; ^5^ Nonagen Bioscience Corporation, Jacksonville, FL 32216, USA; ^6^ Department of Biostatistics, The University of Florida, Gainesville, FL 32611, USA; ^7^ Clinical & Translational Research Program, University of Hawaii Cancer Center, Honolulu, HI 96813, USA; ^8^ Department of Urology, The University of Texas Southwestern Medical Center, Dallas, TX 75390, USA

**Keywords:** urine biomarkers, meta-analysis, urinary bladder, urothelial carcinoma, diagnosis

## Abstract

A 10-plex urine-based bladder cancer (BCa) diagnostic signature has the potential to non-invasively predict the presence of BCa in at-risk patients, as reported in various case-control studies. The present meta-analysis was performed to re-evaluate and demonstrate the robustness and consistency of the diagnostic utility of the 10-plex urine-based diagnostic assay. We re-analyzed primary data collected in five previously published case-control studies on the 10-plex diagnostic assay. Studies reported the sensitivity and specificity of ten urinary protein biomarkers for the detection of BCa, including interleukin 8, matrix metalloproteinases 9 and 10, angiogenin, apolipoprotein E, syndecan 1, alpha-1 antitrypsin, plasminogen activator inhibitor-1, carbonic anhydrase 9, and vascular endothelial growth factor A. Data were extracted and reviewed independently by two investigators. Log odds ratios (ORs) were calculated to determine how strongly the 10-plex biomarker panel and individual biomarkers are associated with the presence of BCa. Data pooled from 1,173 patients were analyzed. The log OR for each biomarker was improved by 1.5 or greater with smaller 95% CI in our meta-analysis of the overall cohort compared with each analysis of an individual cohort. The combination of the ten biomarkers showed a higher log OR (log OR: 3.46, 95% CI: 2.60–4.31) than did any single biomarker irrespective of histological grade or disease stage of tumors. We concluded that the 10-plex BCa-associated diagnostic signature demonstrated a higher potential to identify BCa when compared to any single biomarker. Our results justify further advancement of the 10-plex protein-based diagnostic signature toward clinical application.

## INTRODUCTION

Bladder cancer (BCa) is the second most common genitourinary malignancy in the United States, with 79,030 new cases and 16,870 deaths estimated to occur in 2017 [1]. It is also among the five most common malignancies worldwide [2]. The most common form of BCa in Western countries is urothelial carcinoma, accounting for approximately 90% of all cases [3]. The majority of BCa cases present as non-muscle invasive bladder cancer (NMIBC), which has a 5-year survival rate of >90%. However, once BCa progresses to muscle-invasive bladder cancer (MIBC), the 5-year survival rates do not exceed 50%, and distant metastasis frequently occurs. Metastatic BCa is highly lethal, with a 5-year survival rate of <15% and an estimated median survival of 12 to 14 months [4]. Therefore, early identification, both at the initial diagnosis and at recurrence, is crucial [5].

BCa detection and diagnosis require cystoscopy and bladder biopsy, which are unpleasant and costly procedures. Although NMIBC can be treated with transurethral resection (TUR) with an excellent survival outcome, this method is associated with an intravesical recurrence rate of approximately 70% within two years after TUR [6]. This is the highest recurrence rate among any type of tumor [7]. Therefore, NMIBC patients must be monitored for recurrence, which requires repeat cystoscopies. The high recurrence rates as well as lengthy treatment regimens have caused BCa to be one of the most costly malignancies to manage on a per-patient basis [8]. With an accurate urine biomarker, the number of cystoscopy would be reduced. Thus, there is an urgent need to develop novel diagnostics that are less invasive and less expensive without compromising accuracy for both initial detection and surveillance for BCa.

Recent advancements in proteomics technology have promoted discovery of novel protein markers and the number of published reports on urine-based biomarkers has dramatically increased with reported sensitivity ranges from 52% to 97%, and specificity from 43% to 100% for individual biomarkers (Table 1) (modified from D’Costa and colleagues [9]). Despite these efforts, single use of existing urinary biomarkers is not accurate enough to replace the most widely used urine-based assay, voided urinary cytology (VUC), which has a low sensitivity (range: 13–75%, median 35%) [10].

**Table 1:** Reported sensitivity and specificity of urine-based single protein biomarkers for the detection of bladder cancer

Protein name	Sensitivity (%)	Specificity (%)	Cancer (n)	Control (n)	Ref.
Alpha-1-anti-trypsin	74	80	54	46	[32]
Alpha-1-anti-trypsin	71	72	102	206	[40]
Angiogenin	66	75	50	40	[41]
Apolipoprotein A1	95	92	49	37	[42]
Apolipoprotein A4	79	100	110	66	[18]
AMFR	84	75	45	62	[43]
BIGH3	93	80	30	15	[44]
Calprotectin	80	93	46	40	[45]
Cathepsin B	56	56	122	107	[46]
Cathepsin L	71	75	122	107	[46]
CCL18	70	68	102	206	[40]
CD147	97	100	30	15	[44]
CEACAM1	74	95	95	82	[47]
Clusterin	68	61	68	61	[48]
Clusterin	70	83	50	40	[41]
Coronin-1A	67	100	110	66	[18]
CXCL1	72	95	95	30	[49]
CXCL1	56	84	43	43	[50]
CYFRA21-1	79	89	82	70	[51]
CYFRA21-1	81	97	86	76	[52]
CYFRA21-1	70	43	125	321	[53]
CYFRA21-1	97	67	48	80	[54]
DJ1	83	100	110	66	[18]
EN2	82	75	466	55	[55]
FDP	52	91	57	139	[56]
Fibronectin	91	88	75	55	[57]
Fibronectin	72	82	126	41	[58]
Prothrombin	71	75	76	80	[17]
Reg-1	81	81	23	48	[59]
Semenogelin-2	67	80	110	66	[18]
Stathmin-1	90	87	30	15	[44]
Telomerase	70	99	57	139	[56]
Telomerase	83	89	73	37	[60]
g-synuclein	88	90	110	66	[18]

Recent publications have proposed panels of protein biomarkers for the detection of BCa [11–19]. Chen and colleagues conducted a case-control study to test diagnostic performance of 63 urinary proteins found in their earlier iTRAQ study [17]. They developed a 6-peptide panel that yielded an AUC of 0.814, with a 76.3% positive predictive value, and a 77.5% negative predictive value. Kumar and colleagues developed a panel of five urinary proteins [18]. Both ELISA and Western blot (WB) assays yielded an AUC of 0.9 or more. Particularly, their WB-based assay showed more than 90% sensitivity with an almost 100% specificity. In another study, Theodorescu *et. al.* obtained polypeptide patterns in urine samples using capillary-electrophoresis-coupled mass spectrometry. From signatures of polypeptide mass, they established a model for predicting the presence of BCa at any stage [20] or muscle-invasive disease [21].

In a more recent report, Frantzi and colleagues developed two biomarker panels: one that contained 116 peptides and one that contained 106 peptides [19]. The authors validated the diagnostic performance of the panels using independent cohorts, showing area under the curve (AUC) values of 0.87 and 0.75 for detecting primary and recurrent BCa, respectively. They also demonstrated that the combination of their model with VUC exhibited superior diagnostic accuracy compared with the performance of either test alone. These findings further support the results demonstrating that the multiplex urine-based biomarker panel has superior diagnostic performance compared with single protein markers. Further analyses incorporating these other promising multiplex assays as well as VUC and UroVysion® will be warranted in the future studies.

While the concept that a panel of biomarkers is preferable to single biomarkers is well supported, such marker panels have not widely been developed and implemented in the clinic. In previous studies designed to establish and validate a multiplex urinary immunoassay for BCa detection [11–16, 22], we have examined approximately 1,300 urine samples. This series of studies identified a promising multivariate combination of ten urine-based biomarkers: interleukin 8 (IL8), matrix metalloproteinases 9 and 10 (MMP9 and MMP10), angiogenin (ANG), apolipoprotein E (APOE), syndecan 1 (SDC1), alpha-1 antitrypsin (A1AT), plasminogen activator inhibitor-1 (PAI1), carbonic anhydrase 9 (CA9), and vascular endothelial growth factor A (VEGFA) [23]. In the present study, we conducted a meta-analysis to re-evaluate and demonstrate the diagnostic performance of our 10-biomarker panel.

## RESULTS

### Study selection

We initially selected five studies that our group previously published on the diagnostic abilities for BCa detection of the following urinary biomarkers: ANG, APOE, A1AT, CA9, IL8, MMP9, MMP10, PAI1, SDC1, and VEGF [11–15]. We made an additional systematic search (see Materials and Methods section) but found no other study that met our criteria for the purpose of evaluating diagnostic ability of the 10-plex urinary biomarker panel. Adequacy of the study quality was confirmed using The Newcastle-Ottawa Scale (NOS) [24, 25], while the reporting of each study was evaluated according to Standards for Reporting of Diagnostic Accuracy (STARD) criteria [26, 27].

### Data extraction and categorization

**Figure 1:**
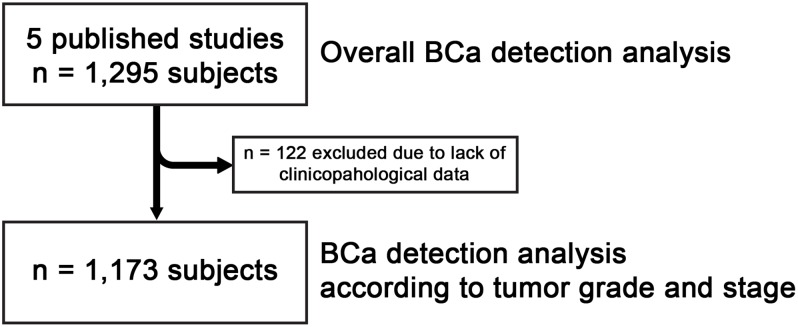
Study subjects for the present analyses

Data extraction from primary data of the five studies [11–15] was conducted independently by 2 investigators (N.M. and T.K.), and categorization was validated in the presence of the moderator (O.O.). Data pooled from the five reports [11–15] consisted of 1,295 patients (Table 2), including 247 females and 1048 males that consisted of clinicopathological and normalized molecular data. The study cohorts were mutually exclusive and there was no overlap in study subjects between the study. Data from these 1,295 patients were analyzed for overall BCa detection. Then 122 patients from Goodison 2012 [11] were excluded due to lack of histological grade or disease stage and data from the remaining 1,173 patients were analyzed for BCa detection according to tumor grade or stage, as depicted in Figure 1. This was accomplished by review of the original data.

**Table 2:** Summary of bladder cancer cases and controls in each cohort analyzed in the present study

Cohort		n	Male (%)	Median age (Years)	HG tumor (%)	MIBC (%)	Assay method
Goodison 2012 [11]	Case Control	64 63	86 87	69.5 60	86.0	58.7	ELISA
Rosser 2013 [12]	Case Control	102 206	82 74	69 56	62.7	40.2	ELISA
Chen 2014 [13]	Case Control	183 137	84 72	69 65	55.7	16.4	ELISA
Shimizu 2016 [14] Cohort 1	Case Control	29 33	86 82	68 50	86.2	44.8	Multi-Array
Cohort 2	Case Control	100 100	82 81	70 50.5	79.0	42.0	Multi-Array
Goodison 2016 [15]	Case Control	211 67	87 79	75 70	58.8	19.4	Multi-Array

ELISA, enzyme-linked immunosorbent assay; HG, high-grade; MIBC, muscle-invasive bladder cancer.

### Meta-analysis

**Figure 2:**
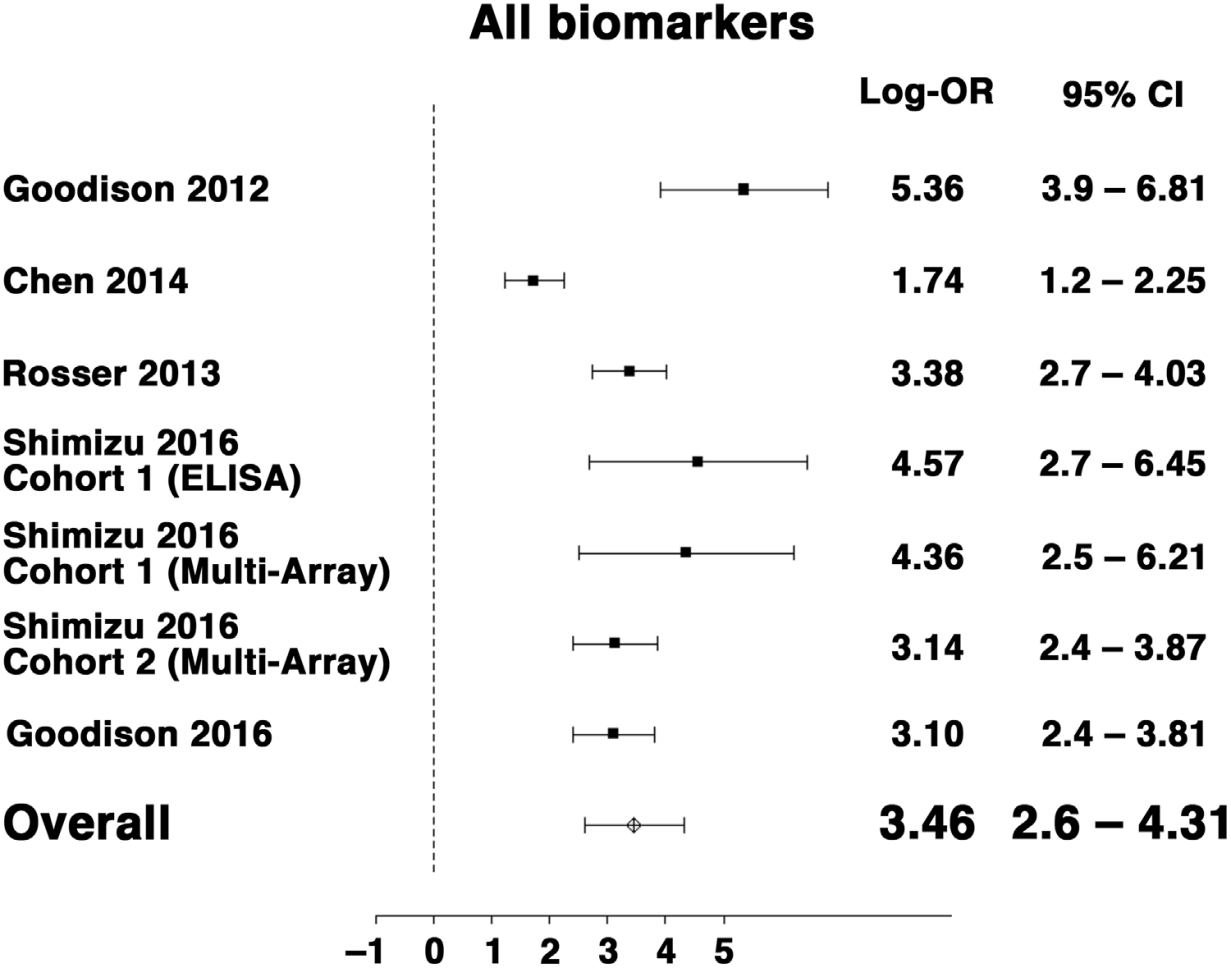
Forest plot for random-effects meta-analysis of the association between multiplex BCa biomarkers and the outcome of detecting BCa from voided urines (any stage or grade, n = 1,295) Effect sizes are expressed as odds ratios. Studies are represented by symbols whose area is proportional to the weight of the study in the analysis.

**Figure 3:**
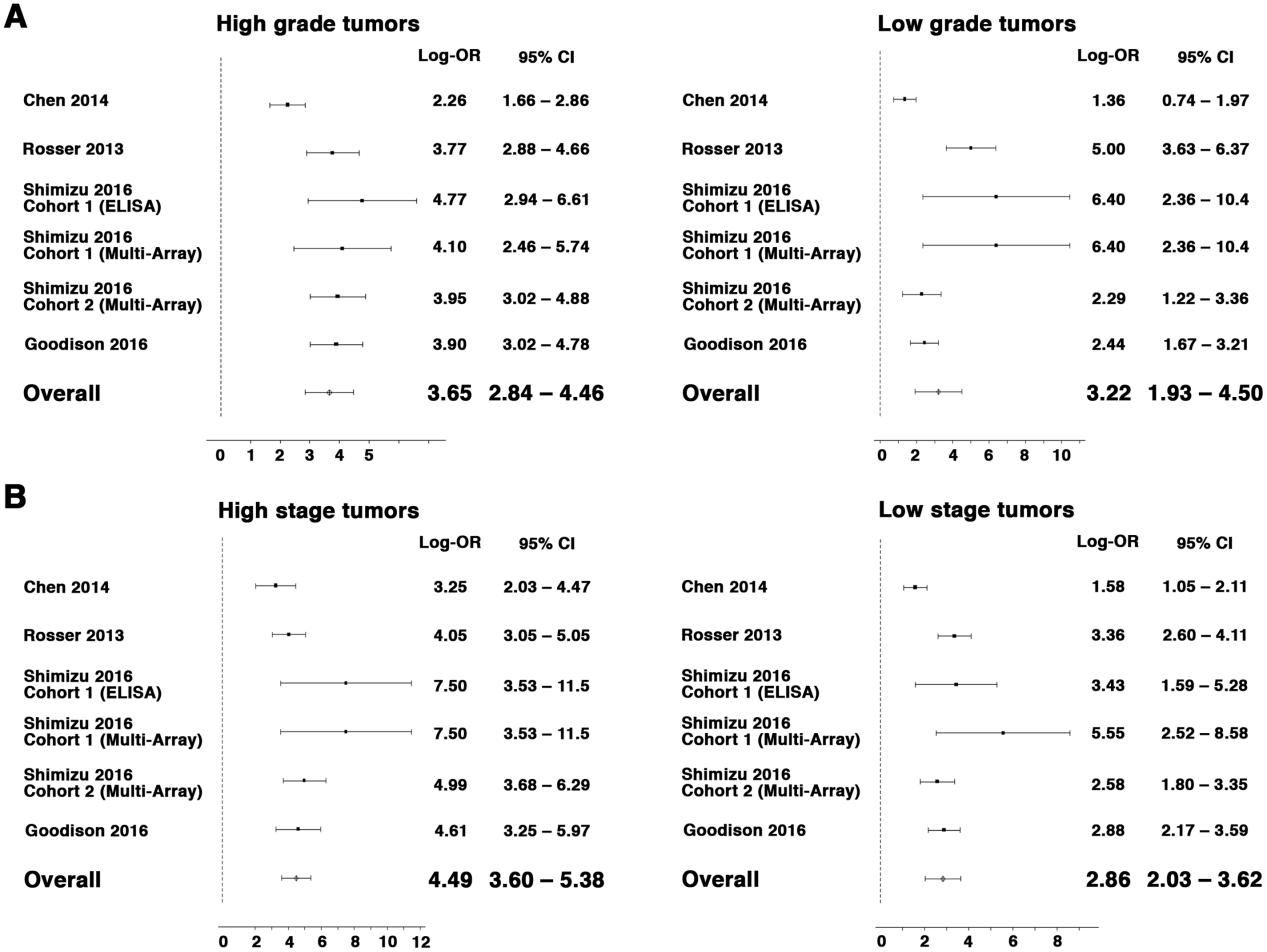
Forest plot for random-effects meta-analysis of the association between multiplex BCa biomarkers and tumor grade (**A**, high-grade, left panel, low-grade, right panel) and tumor stage (**B**, T2 or greater stage, left panel and Ta/T1 stage, right panel) (n = 1,173). Effect sizes are expressed as odds ratios. Studies are represented by symbols whose area is proportional to the weight of the study in the analysis.

As shown in Figure 2, the log OR for the combination of the ten urinary protein biomarkers (n = 1,295, log OR: 3.46, 95% CI: 2.60–4.31), ranged from 1.74 to 5.36 depending on the report confirming the utility of the ten protein biomarkers in detecting BCa from a urine sample. Furthermore, advantage of the combination of the ten urinary protein biomarkers was robust when it was analyzed with regard to high-grade (log OR: 3.65, 95% CI: 2.84–4.46) and low-grade (log OR: 3.22, 95% CI: 1.93–4.50) disease as well as with regard to high stage (T2 or greater, log OR: 4.49, 95% CI: 3.60–5.38) and low stage (Ta/T1, log OR: 2.86, 95% CI: 2.03–3.62) disease (Figure 3).

**Figure 4:**
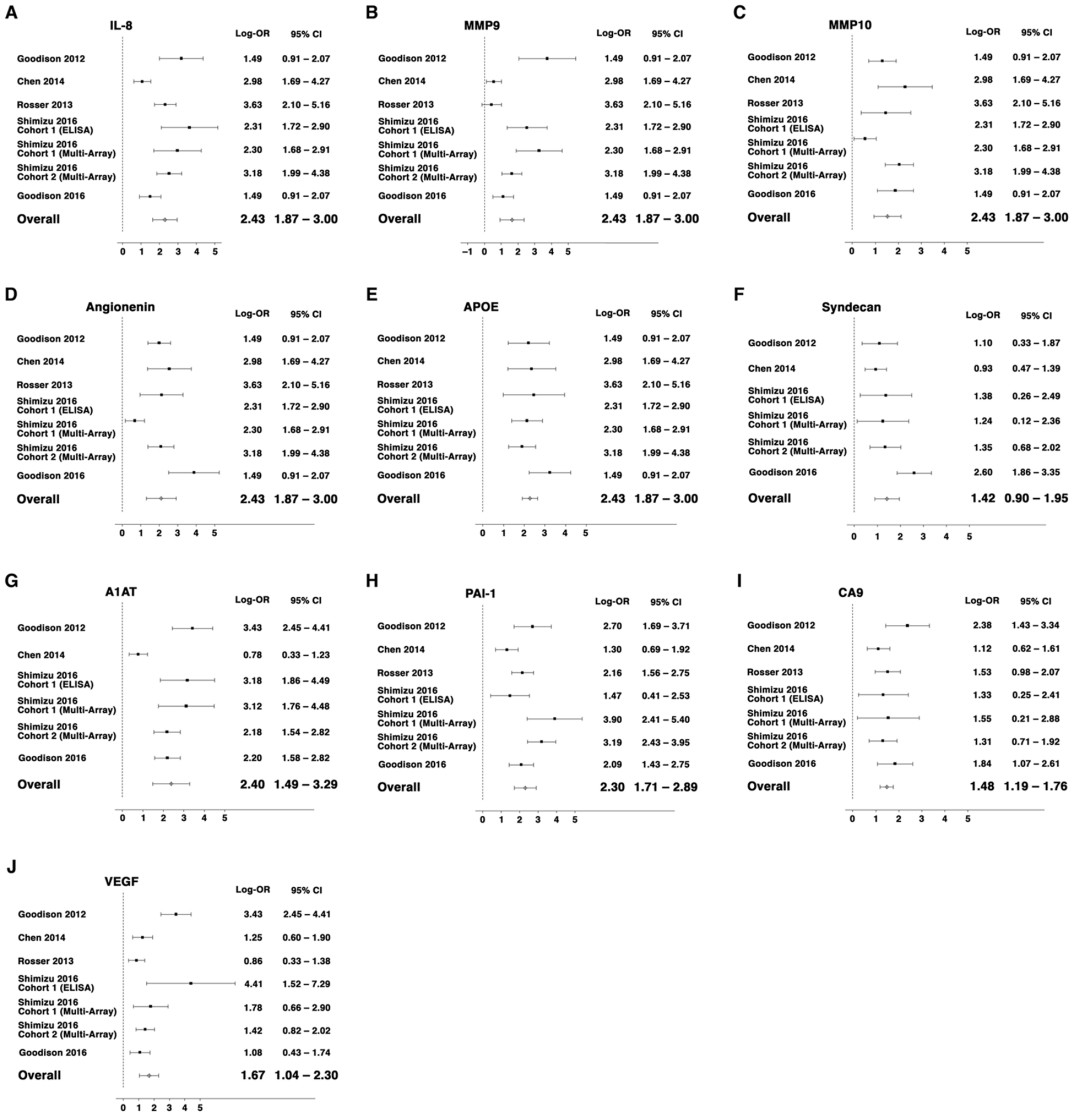
Forest plots for random-effects meta-analysis of the association between individual BCa biomarkers and the outcome of detecting BCa from voided urines (any stage or grade, n = 1,295) Effect sizes are expressed as odds ratios. Studies are represented by symbols whose area is proportional to the weight of the study in the analysis.

The log OR for each biomarker was improved by 1.5 or greater with smaller 95% CI in our meta-analysis of the overall cohort compared with each analysis of an individual cohort. A1AT (log OR: 2.40, 95% CI: 1.49–3.29), PAI1 (log OR: 2.30, 95% CI: 1.71–2.89) and IL-8 (log OR: 2.29, 95% CI: 1.63–2.96) showed the highest log OR, while MMP10 (log OR: 1.36, 95% CI: 0.87–1.85) showed the lowest (Figure 4).

## DISCUSSION

A successful meta-analysis allows compiling data from previous studies, thus elevating the robustness and the level of evidence from the single studies. In the present study, indeed, we demonstrated that the combination of 10 urine-based biomarkers was more strongly associated with BCa than was any single biomarker. The finding is in agreement with other studies. For example, other investigators have employed capillary electrophoresis coupled with mass spectrometry (CE-MS), followed by support vector machine algorithms [28], to develop diagnostic models for BCa [19–21] and other diseases [29, 30]. In these previous reports, panels of multiple protein biomarkers exhibited diagnostic accuracy superior to any single protein biomarker.

The urine-based protein biomarkers analyzed in the present study were originally established as a panel of 14 protein biomarkers [11] using a bioinformatics approach integrating information from genomics [31] and proteomics [32, 33] analyses. Subsequent studies streamlined this into a panel of 10 protein biomarkers on the ELISA platform [12, 13, 16]. More recently, a custom electrochemiluminescent multiplex platform was developed [14] and validated [15] to facilitate quick and high-throughput analysis of all 10 protein biomarkers simultaneously in a single assay without loss of performance.

Currently, published guidelines recommend that patients presenting with hematuria undergo VUC and examination using cystoscopy [5, 34–36], an invasive, uncomfortable and expensive procedure associated with possible adverse effects. We believe that the employment of a multiplex, proteomic urinary assay can reduce the need to subject large numbers of patients who do not have BCa to uncomfortable and expensive cystoscopic examinations and thus ‘rule-in’ patients who require a more thorough evaluation. The 10-plex proteomic assay evaluated in this study is currently being tested in a phase III study in the US for both detection and surveillance.

As for influence of other diseases, the urine-based protein biomarkers have been already assessed in patients with other genitourinary malignancies and renal disorders, *e.g.*, prostate cancer, kidney cancer and chronic kidney disease. There was limited overlap of the biomarkers in prostate cancer (only IL-8 was elevated) and kidney cancer (only CA9 and VEGF were elevated). In chronic kidney disease, *i.e.*, GFR < 45 mL/min, significant amounts of proteins were evident in the urine and thus the assay is unable to accurately discriminate if a patient has cancer (data not shown). Urinary tract infection (UTI) is another coincidence that can negatively affect the diagnostic performance of urine-based biomarkers. The present study included 96 subjects with UTI and the 10-plex panel as well as most of the single markers showed better performance with the subjects excluded from the analysis (data not shown). These findings suggest that the 10-plex panel is anticipated to yield an excellent performance in a cohort including those with UTI although it should be applied to those subjects with caution.

Several limitations of this study must be acknowledged. Although targets in all included studies were quantitatively measured, the antibodies used to monitor each urine-based biomarker were not identical among the included studies. The present study did not incorporate detailed data such as race, gender, age, and smoking history, which has been reported to influence diagnostic performance of the multiplex urinary protein panel [22]. Since all included studies were case-control designs, it is unclear whether the diagnostic accuracy will be reproducible in clinically relevant cohorts such as consecutive individuals who are referred with hematuria, or those on post-TUR surveillance for intravesical recurrence of BCa, in which the prevalence of BCa may be different from those in the included studies. It is not clear whether the replacement of cystoscopy by the 10-plex assay is cost-effective or not, since the cost of the 10-plex assay is yet to be determined. Despite these limitations, this study emphasizes the potential of a multiplex urinary protein assay and justifies the advancement of the assay to the next phase of the developmental stages of urinary biomarkers for BCa detection, proposed by the International Bladder Cancer Network [37, 38].

In conclusion, our meta-analysis confirmed significant association between urinary levels of the protein biomarkers and BCa detection. In particular, the combination of the ten biomarkers demonstrated a higher potential for detection of BCa than did any single biomarker. The study has justified further advancement of the multiplex urinary protein biomarker assay toward clinical application as a noninvasive method of detecting BCa in our daily practice. However, further validation steps including analyses of consecutive patients are needed before clinical adoption [39].

## MATERIALS AND METHODS

### Database search

An additional search was conducted using Medline and Embase using the following urinary biomarkers for BCa in the search bar: ANG, APOE, A1AT, CA9, IL8, MMP9, MMP10, PAI1, SDC1, and VEGF. The following additional filters were selected: “Publication dates from January 1, 2012 to December 31, 2016” and studies in “Humans.” Studies assessing the biomarker panel in subjects for the purpose of tumor surveillance were excluded. Similarly, studies not describing the 10 biomarkers in a multiplex format for the diagnosis of BCa were excluded. Eventually no article was found in addition to the five studies that we initially selected.

### Meta-analysis

We performed a meta-analysis using a random-effect model followed by multivariable-pooled analysis of the molecular data using the weighted least-squares method to account for size effects. Random-effect meta-regression models (linear mixed models) were used to assess the relationship between the estimates and the outcome (BCa *vs.* no BCa), adjusted for other potential confounders and/or mediators, as appropriate. Note that the weighted least-squares method under the multivariable-pooled analysis can better overcome small-sample-size bias, whereas the random-effect meta-regression model can better overcome between- and within-study heterogeneity. Both methods were applied to generate the most robust results. Statistical analyses were performed using R version 3.2.3 and reviewed by Y.D.
